# Clinical characteristics of headache after vaccination against COVID-19 (coronavirus SARS-CoV-2) with the BNT162b2 mRNA vaccine: a multicentre observational cohort study

**DOI:** 10.1093/braincomms/fcab169

**Published:** 2021-07-23

**Authors:** Carl H Göbel, Axel Heinze, Sarah Karstedt, Mascha Morscheck, Lilian Tashiro, Anna Cirkel, Qutayba Hamid, Rabih Halwani, Mohamad-Hani Temsah, Malte Ziemann, Siegfried Görg, Thomas Münte, Hartmut Göbel

**Affiliations:** 1 Department of Neurology, University Hospital Schleswig-Holstein, Lübeck, Germany; 2 Kiel Migraine and Headache Centre, Kiel, Germany; 3 College of Medicine, University of Sharjah, Sharjah, United Arab Emirates; 4 College of Medicine, King Saud University, Riyadh, Kingdom of Saudi Arabia; 5 Institute of Transfusion Medicine, University Hospital Schleswig-Holstein, Lübeck, Germany

**Keywords:** COVID-19, mRNA vaccine, side-effects, headache, International Classification of Headache Disorders

## Abstract

The novel coronavirus SARS-CoV-2 causes the infectious disease COVID-19. Newly developed mRNA vaccines can prevent the spread of the virus. Headache is the most common neurological symptom in over 50% of those vaccinated. Detailed information about the clinical characteristics of this form of headache has not yet been described. The aim of the study is to examine in detail the clinical characteristics of headaches occurring after vaccination against COVID-19 with the BNT162b2 mRNA COVID-19 vaccine for the first time. In a multicentre observational cohort study, data on the clinical features and corresponding variables were recorded using a standardized online questionnaire. The questionnaire was circulated to 12 000 residential care homes of the elderly as well as tertiary university hospitals in Germany and the United Arab Emirates. The primary outcomes of this study are the clinical features of headache after vaccination. Comorbidities, treatment with medication and sociodemographic variables are also analysed. A total of 2349 participants reported headaches after vaccination with the BNT162b2 mRNA COVID-19 vaccine. Headaches occur an average of 18.0 ± 27.0 h after vaccination and last an average duration of 14.2 ± 21.3 h. Only 9.7% of those affected also report headaches resulting from previous vaccinations. In 66.6% of the participants, headache occurs as a single episode. A bilateral location is indicated by 73.1% of the participants. This is most often found on the forehead (38.0%) and temples (32.1%). A pressing pain character is indicated by 49.2% and 40.7% report a dull pain character. The pain intensity is most often moderate (46.2%), severe (32.1%) or very severe (8.2%). The most common accompanying symptoms are fatigue (38.8%), exhaustion (25.7%) and muscle pain (23.4%). Headaches after COVID-19 vaccination show an extensive complex of symptoms. The constellation of accompanying symptoms together with the temporal and spatial headache characteristics delimit a distinctive headache phenotype.

## Teaser

Göbel et al. studied 2349 patients who suffered headaches after their COVID-19 vaccination with the BioNTech/Pfizer mRNA vaccine. The headaches showed an extensive complex of symptoms. The constellation of accompanying symptoms together with the temporal and spatial headache characteristics delimit a distinctive phenotype that allows differentiation from other headache forms.

## Graphical Abstract

**Graphical Abstract:**
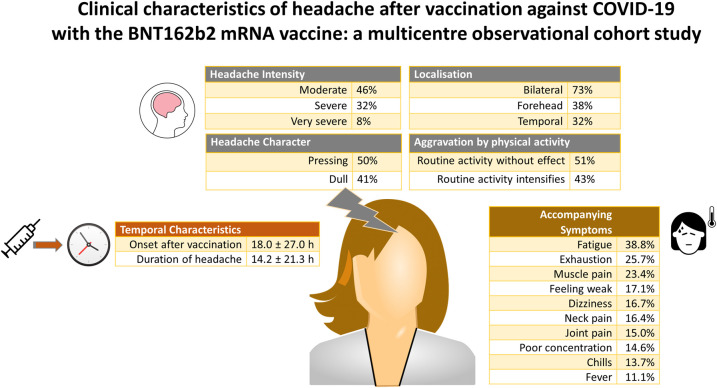

## Introduction

The novel coronavirus SARS-CoV-2 causes the infectious disease COVID-19.^1^ The disease has developed into a global pandemic since the beginning of 2020.^2^ While the infection may present with mild to moderate intensity in some patients, severe forms may result in pronounced dyspnoea, respiratory failure and ultimately death.^3^ In addition to behavioural interventions such as social distancing, newly developed vaccines that are available since the second half of 2020 are the most important countermeasures to prevent the spread of the virus.^4^ According to the data available, the vaccines approved by regulatory authorities to date have a positive efficacy and side-effect profile.^5–10^ Worldwide, there are currently 66 vaccines in clinical and 176 in pre-clinical development.^4^

Using the example of the BNT162b2 mRNA COVID-19 vaccine, the most commonly reported adverse events in vaccinees from 16 years of age are injection site reactions (84.1%), fatigue (62.9%), headache (55.1%), muscle pain (38.3%), chills (31.9%), joint pain (23.6%) and fever (14.2%).^11^ These are usually mild or moderate and remit within a few days after vaccination.^5^

According to the data available, the most common neurological symptom is headache which occurs in over 50% of those vaccinated.^5^ Detailed information about the clinical characteristics of this headache has not yet been described. Regarding headache frequency, it is known that after vaccination with the first dose, mild headaches occur in 27.4%, moderate headaches in 13.4% and severe headaches in 1.0%. After the second vaccination, mild headaches occur in 25.6%, moderate headaches in 22.9% and severe headaches in 3.2%.^11^

The International Classification of Headache Disorders, 3rd Edition^12^ does not yet classify any vaccination-associated headaches and also does not list any diagnostic criteria for such headaches. In addition, the mRNA COVID-19 vaccines are used for the very first time in the context of vaccinations.^5^ The detailed clinical phenotype of the vaccination-associated headache is not known to date. This includes, amongst others, the latency between the vaccination and occurrence of the headache, its temporal course, the headache character, the pain localization, the accompanying symptoms, any pain-modulating factors and possible comorbid conditions.

The currently undergoing global vaccination campaign against COVID-19 allows this new form of headache to be studied in parallel with the vaccinations. The aim of this study is therefore to examine in detail the clinical characteristics of headaches occurring after vaccination against COVID-19. This report analyses headaches occurring after vaccination with the BNT162b2 mRNA COVID-19 vaccine.^5^^,^^11^

## Methods

### Study design and setting

The study is a continuous ongoing multicentre observational cohort study accompanying the COVID-19 vaccination campaign. Ethical approval for this study was obtained from the ethics committee of the University of Kiel (D403/21). All study information and patient consent forms were approved by the ethics committee. The study was performed in accordance with the principles of the Declaration of Helsinki of 1964 and its subsequent revisions.

With a publicly available online questionnaire, specific aspects of the headache phenotype and related variables are collected (questionnaire link: https://schmerzklinik.de/impfung). The questionnaire is available in different language versions. It contains 43 questions about the clinical characteristics of headaches after COVID-19 vaccination. The questions are divided into the following groups: type of vaccine used, occurrence of headaches after vaccination, possible headaches after previous vaccinations against other diseases, temporal parameters of the headache, headache localization, headache characteristics, headache intensity, accompanying symptoms, previous history of headaches, other comorbid diseases and socio-demographic variables.

The study started in Germany. In this report, we present data that were collected from the vaccinated participants in the period from 8 January 2021 to 26 February 2021. According to the German ordinance on the entitlement to vaccination against the SARS-CoV-2 coronavirus, vaccinations are given with the highest priority to people over the age of 80, as well as to inhabitants and employees at residential care homes for the elderly. Healthcare workers at a very high risk of exposure to the SARS-CoV-2 coronavirus were also given the highest priority. Since direct contact with the vaccinees is not possible due to data protection regulations, the management of the residential care homes for the elderly was contacted by email and asked to pass on the information about the study to the residents as well as staff. A total of 12 000 residential care homes in Germany were contacted. In addition, the departments responsible for organizing the vaccinations at all university hospitals in Germany and the United Arab Emirates were contacted. They were asked to inform the employees about the study as part of the ongoing vaccination campaign. Furthermore, attention was drawn to the study via the institutes’ websites and social media. In Germany, vaccination started with the BNT162b2 mRNA COVID-19 vaccine.^5^^,^^11^ This evaluation reports the clinical headache characteristics of those patients who received this vaccine.

### Data collection

The data on the clinical features and corresponding variables were recorded using a standardized online questionnaire. The answers were collected in an online database. At the beginning of the questionnaire, subjects were informed that data were collected anonymously in compliance with the recommendations of the ethics committee. They were also informed that due to the anonymization, it is not possible to revoke participation in the study after any answers have been sent.

### Outcomes

The primary outcomes of this study are the clinical features of headache after vaccination against COVID-19 with the BNT162b2 mRNA COVID-19 vaccine. Comorbidities, treatment with medication and sociodemographic variables are also analysed.

### Bias and missing data

People being cared for in residential care homes for the elderly as well as medical staff in tertiary university hospitals are overrepresented in this study compared to the general population, as they belong to the groups with the highest vaccination priority in Germany. The study is unable to analyse headache characteristics from people who did not voluntarily participate in this study. Missing data were not assumed for this descriptive analysis. Complete data were not available for all variables, so the denominators differ between individual analyses.

### Statistical analysis

Continuous variables are represented as the arithmetic mean and standard deviations. Categorical variables are presented as frequency (%) unless otherwise stated. The *t*-test was used to statistically analyse continuous variables for significant differences. The statistical information is based on non-missing data. The 5% significance level (alpha = 0.05) was considered to be statistically significant. The statistical analyses were carried out using SPSS 27.

### Data availability

The data that support the findings of this study are available from the corresponding author upon reasonable request.

## Results

### Participants

**Figure 1:**
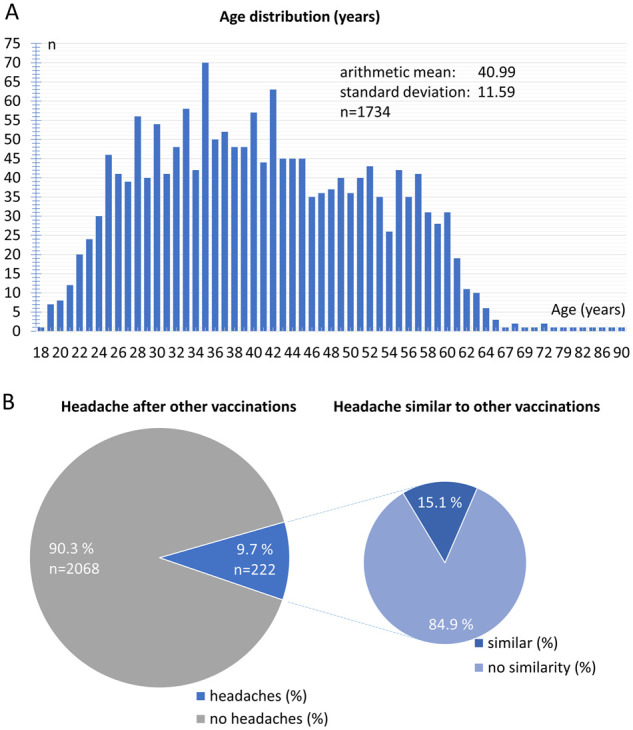
**Demographic data.** (**A**) Age distribution (years) of participants. (**B**) Relative frequency distribution of headaches after previous vaccinations.

Between 8 January 2021 and 26 February 2021, 2349 participants answered yes to the question of whether headaches had occurred after vaccination against COVID-19 with the BNT162b2 mRNA vaccine (Table 1). The participants consisted of 74.3% women and 25.7% men. At the time of vaccination, the mean age was 41.0 ± 11.6 years, the median was 40 years and the range was 18–90 years (Fig. 1A). The mean height was 171.4 ± 10.8 cm, and the mean body weight was 78.2 ± 19.6 kg. Of the participants, 42% had a past history of a primary headache. Two hundred seventy participants stated a previous diagnosis of migraine, 208 participants of both migraine and tension-type headache, 497 participants of tension-type headache and 21 participants of cluster headache. A history of other headache diagnoses was reported by 144 participants. The study does not differentiate whether headaches occur after the first or second vaccination dose. Since the survey began at the start of the vaccination campaign, it is usually a headache after the first vaccination dose.

**Table 1:** Demographic data of patients

Demographic data	
*n*	2349
Sex—*n* (%)	
Female	1289 (74.3%)
Male	446 (25.7%)
Age at vaccination (years)	
Arithmetic mean	40.99
Standard deviation	11.59
Median	40
Range	18–90
Height (cm)	171.4 ± 10.8
Body weight (kg)	78.2 ± 19.6

### Headaches following other vaccinations

Of the participants, 90.3% stated that they had not experienced any headaches following other vaccinations in the past (Fig. 1B). Merely 9.7% of the participants reported that they had also experienced headaches after previous vaccinations. Within this group, 84.9% reported that the headache after COVID-19 vaccination was not similar to previous headaches after other vaccinations. Only 15.1% of the participants reported similar headaches after their COVID-19 vaccination compared to other vaccinations.

### Temporal parameters of headache after vaccination against COVID-19

**Figure 2:**
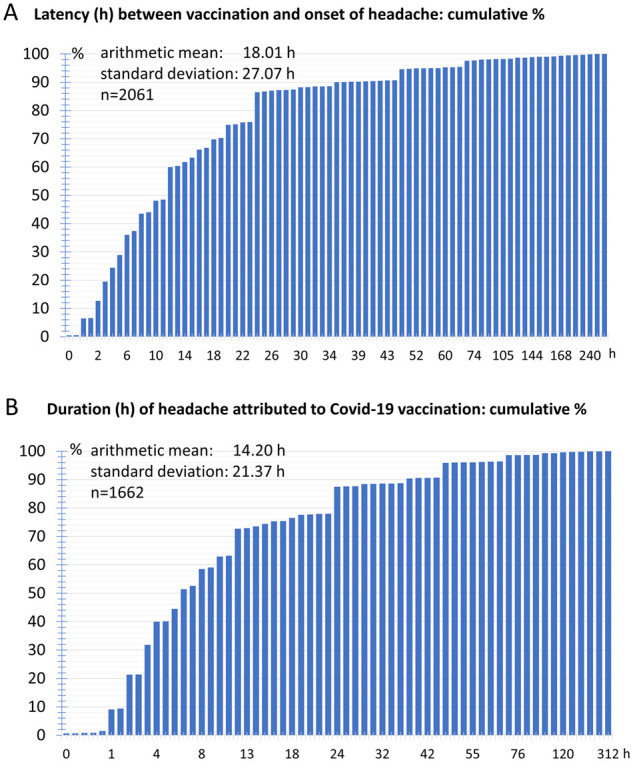
**Temporal characteristics of headaches**. (**A**) Cumulative frequency distribution of latency between vaccination and onset of headache. (**B**) Cumulative frequency of duration of headache attributed to vaccination.

The latency between vaccination against COVID-19 and the occurrence of headaches was on average 18.0 ± 27.0 h. More than half of the participants perceived the headache after less than 10 h and 80% within 24 h after the vaccination. In less than 10% of the participants the headaches only began more than 2 days after the vaccination (Fig. 2A).

The mean headache duration was 14.2 ± 21.4 h. In 50% of the participants the headache duration was less than 6 h and in 80% less than 22 h. The headache lasted longer than 36 h in only 10% of the participants. The maximum headache duration reported in a single case was 312 h (Fig. 2B).

Of the participants, 66.6% reported that the headache occurred continuously as a single episode without interruption and 33.4% answered that the headache occurred in multiple phases. The average duration of the headache-free interval between the individual phases was 4.9 ± 12.3 h.

### Headache location and radiation

**Figure 3:**
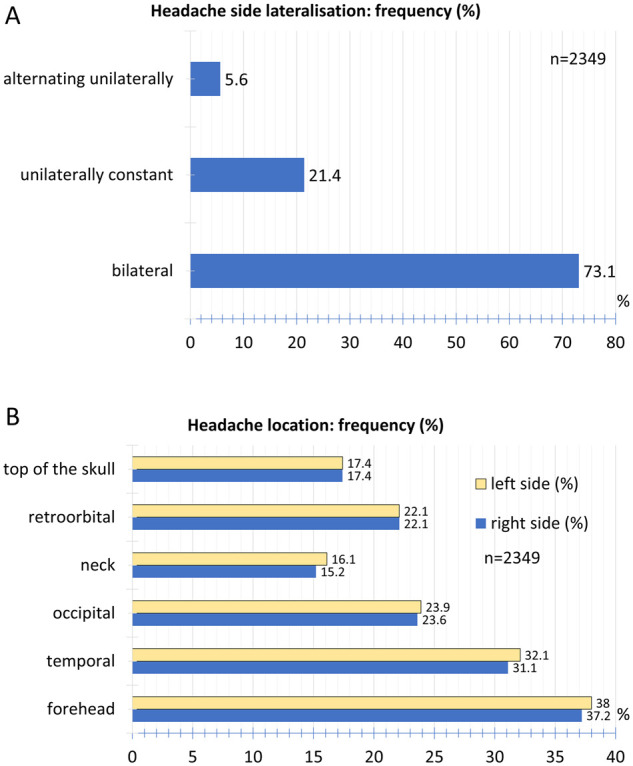
**Spatial characteristics of headaches**. (**A**) Relative frequency distribution of headache side lateralization. (**B**) Relative frequency distribution of headache location.

The headache occurred bilaterally in 73.1% of the participants. A non-varying unilateral pain was reported by 21.4% of the participants. An alternating one-sided headache was indicated by 5.6% of the participants (Fig. 3A).

**Figure 4:**
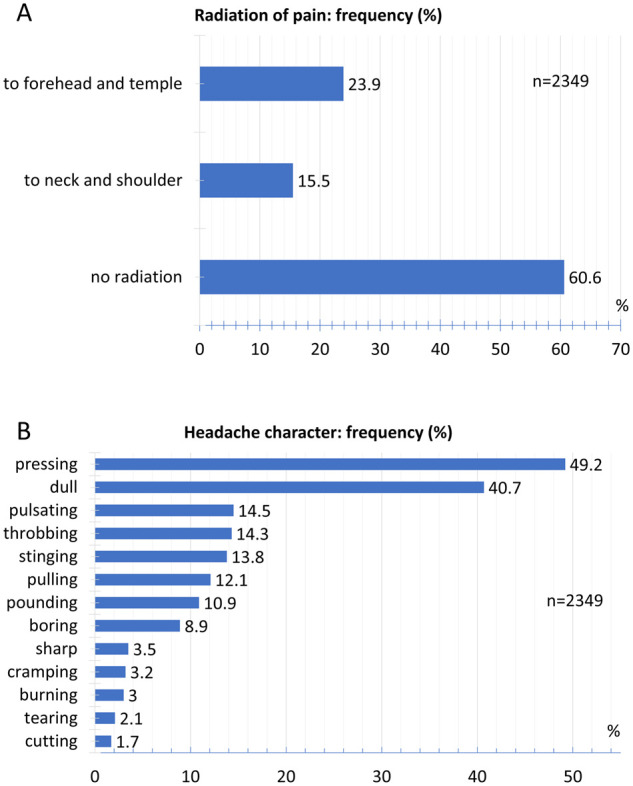
**Radiation and headache character**. (**A**) Relative frequency distribution of radiation of pain. (**B**) Relative frequency distribution of headache character.

The most frequent headache location was found in the forehead area (38.0% left, 37.2% right) followed by the temple region (32.1% left, 31.1% right) and the back of the head (23.9% left, 23.6% right). Retroorbital pain occurred in 22.1% on the left and 22.1% on the right. Pain on the top of the skull was indicated by 17.4% on the left and 17.4% on the right, the neck by 16.1% on the left and 15.2% on the right (Fig. 3B). Of the participants, 60.6% reported no radiation of pain, 15.5% of pain to the neck and shoulder and 23.9% to the forehead and temples (Fig. 4A).

### Headache character

Of the participants, 49.2% reported a pressing and 40.7% a dull pain character (Fig. 4B). With a lesser frequency, 14.5% of the patients reported a pulsating pain and 14.3% reported a throbbing pain character. A stinging pain was mentioned by 13.8%, a pulling pain by 12.1% and a pounding pain by 10.9% of the participants. Other pain characters were documented by less than 10% of the participants.

### Headache intensity

**Figure 5:**
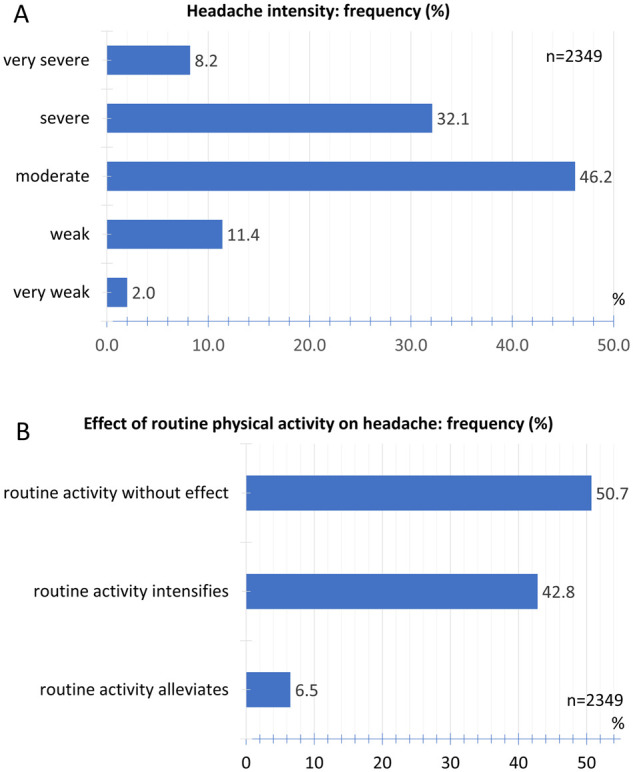
**Intensity and effect of physical activity**. (**A**) Relative frequency distribution of headache intensity. (**B**) Relative frequency distribution of effect of routine physical activity on headache.

The headache after COVID-19 vaccination with the BNT162b2 mRNA COVID-19 vaccine was described as moderate by 46.2% and severe by 32.1%. Of the participants, 8.2% reported a very severe headache. A very mild headache was reported by 2.0% and a mild headache by 11.4% of the participants (Fig. 5A).

Of the participants, 50.7% stated that routine physical activity had no effect on headache intensity. 42.8% reported that routine physical activity worsened the headache, 6.5% of the participants stated that routine physical activity relieved the headache (Fig. 5B).

### Accompanying symptoms

**Figure 6:**
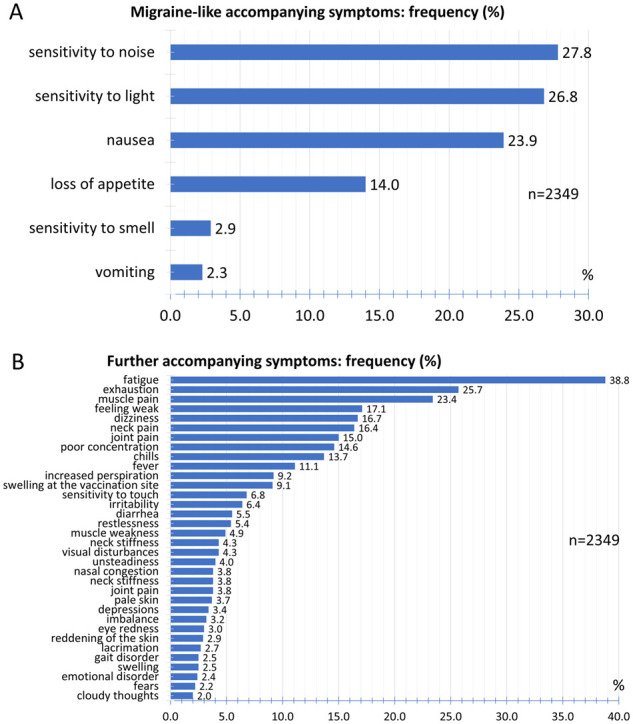
**Accompanying symptoms**. (**A**) Relative frequency distribution of migraine-like accompanying symptoms. (**B**) Relative frequency distribution of further accompanying symptoms.

Regarding accompanying symptoms, migraine-typical accompanying symptoms and other accompanying symptoms were evaluated separately. Sensitivity to noise (27.8%), sensitivity to light (26.8%) and nausea (23.9%) were the most common symptoms in the group of symptoms typical of migraine. Loss of appetite (14.0%), hypersensitivity to smell (2.9%) and vomiting (2.3%) were less common (Fig. 6A).

The most common further accompanying symptoms were fatigue (38.8%), exhaustion (25.7%) and muscle pain (23.4%) (Fig. 6B). With a frequency between 10% and 17.1%, the participants reported neck pain, joint pain, poor concentration, chills and fever. Increased perspiration, swelling at the vaccination site, sensitivity to touch, irritability, diarrhoea and restlessness were reported by the participants with a frequency between 5% and 10%. Further accompanying symptoms with a frequency less than 5% are listed in Fig. 6B.

### Past medical history

**Figure 7:**
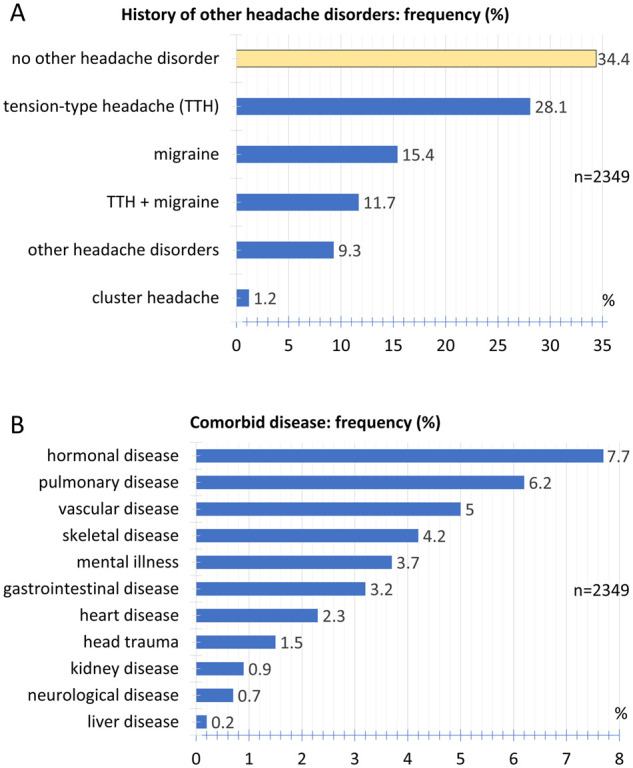
**Comorbidities of headaches**. (**A**) Relative frequency distribution of history of other headache disorders. (**B**) Relative frequency distribution of comorbid diseases.

Of the participants, 34.4% stated that they had no prior history of headache disorders. As the most common headache disorder, 28.1% of participants reported a tension-type headache. 15.4% documented a migraine and 11.7% the coexistence of both tension-type headache and migraine. Cluster headaches were reported by 1.2%. 9.3% of participants reported a history of other headache disorders (Fig. 7A).

The frequencies of other comorbid diseases are listed in Fig. 7B. The most common ones are hormonal, such as diseases of the thyroid or pituitary gland and the pancreas (7.7%), pulmonary (6.2%) and vascular disorders (5.0%).

### Treatment

**Figure 8:**
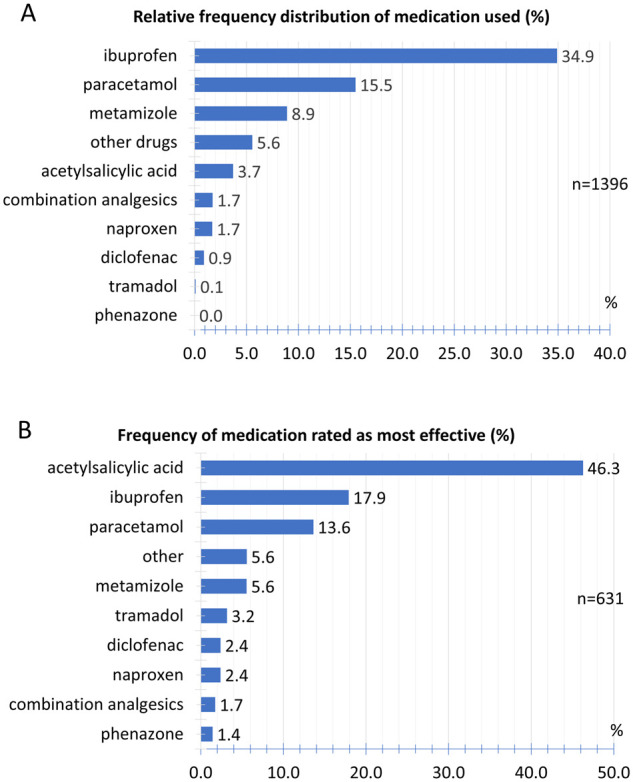
**Treatment of headaches**. (**A**) Relative frequency distribution of medication used. (**B**) Relative frequency of medication rated as most effective.

For the acute treatment of headaches, 34.9% of the participants used ibuprofen, followed by paracetamol (15.5%) and metamizole (8.9%). Other drugs used are listed in Fig. 8A.

When participants took multiple drugs to treat the headache, they were asked to rank for the most effective drug. Figure 8B shows the relative frequency with which an active ingredient was rated as the most effective drug.

### Association with pre-existing headache disorders

To investigate a possible connection between pre-existing headache disorders and headache occurring after COVID-19 vaccination, the effect of pre-existing headache illnesses on quantitative parameters of headaches after COVID-19 vaccination was investigated (Table 2). In participants who reported a history of migraine, there was no effect on the time between the vaccination and the onset of the headache compared to participants who had no primary headache. However, the duration of the headache after the COVID-19 vaccination was significantly longer in patients with a history of migraine than in participants without primary headache (16.5 ± 20.1 versus 13.0 ± 18.5 h; *P* = 0.008). In addition, migraine patients reported a significantly higher headache pain intensity on the verbal rating scale (VRS 0–5) after COVID-19 vaccination compared to participants who had no history of migraine (3.5 ± 0.9 versus 3.2 ± 0.9; *P* < 0.001).

**Table 2:** Comparison of patients with pre-existing primary headaches (migraine, tension-type headache or cluster headache) with patients without pre-existing primary headaches with regard to quantitative parameters of headaches attributed to COVID-19 vaccination (arithmetic mean and standard deviation. Statistical analysis using *t*-test)

	Migraine	Tension-type headache	Cluster headache	No primary headaches
Latency between vaccination and onset of headache (h)	18.8 ± 27.0 *n* = 468 *P = 0.595*	15.6 ± 24.6 *n* = 492 ***P = 0.018***	22.7 ± 37.8 *n* = 21 *P = 0.671*	19.7 ± 31.0 *n* = 600
Duration of headache attributed to COVID-19 vaccination (h)	16.5 ± 20.1 *n* = 393 ***P = 0.008***	13.6 ± 24.3 *n* = 366 *P = 0.706*	10.8 ± 12.2 *n* = 21 *P = 0.581*	13.0 ± 18.5 *n* = 490
Intensity of headache attributed to COVID-19 vaccination (VRS 0–5)	3.5 ± 0.9 *n* = 473 ***P < 0.001***	3.3 ± 0.8 *n* = 492 *P = 0.172*	3.2 ± 0.9 *n* = 21 *P = 0.830*	3.2 ± 0.9 *n* = 602

Participants who reported a history of tension-type headache showed a significantly shorter latency between vaccination and onset of headache (15.6 ± 24.6 versus 19.7 ± 31.0; *P* < 0.018). Patients with cluster headache, on the other hand, showed no difference in the time interval between vaccination and the onset of headache as well as the duration of the headache after COVID-19 vaccination and headache intensity compared to participants without primary headache (Table 2).

### Gender differences

The latency between vaccination and onset of headache showed no significant differences between women and men (Table 3). In contrast, the duration of the headache did significantly differ between women and men (14.8 ± 22.2 versus 12.1 ± 14.87 h; *P* = 0.033). There was also a significant difference between women and men with regard to intensity of headache (VRS 0–5) attributed to COVID-19 vaccination (3.4 ± 0.8 versus 3.1 ± 0.8; *P* < 0.001).

**Table 3:** Group comparison of women and men with regard to quantitative parameters of headaches attributed to COVID-19 vaccination (arithmetic mean and standard deviation. Statistical analysis using *t*-test)

	Women	Men	*t*-test
Latency between vaccination and onset of headache (h)	17.6 ± 28.0 *n* = 1283	19.7 ± 26.8 *n* = 445	*P = 0.166*
Duration of headache attributed to COVID-19 vaccination (h)	14.8 ± 22.2 *n* = 1032	12.1 ± 14.9 *n* = 353	***P = 0.033***
Intensity of headache attributed to COVID-19 vaccination (VRS 0–5)	3.4 ± 0.8 *n* = 1289	3.1 ± 0.8 *n* = 446	***P < 0.001***

### Age effect

When the participants were split up into a group of up to 55 years of age and of older than 55 years of age, no significant differences were found with regard to the interval between vaccination and the onset of the headache, as well as the duration of the headache (Table 4). The headache intensity also did not differ between the two groups.

**Table 4:** Age group comparison of participants ≥55 years and <55 years with regard to quantitative parameters of headaches attributed to COVID-19 vaccination (arithmetic mean and standard deviation. Statistical analysis using *t*-test)

	≥55	<55	*t*-test
Latency between vaccination and onset of headache (h)	19.0 ± 24.4 *n* = 270	18.0 ± 28.3 *n* = 1457	*P = 0.576*
Duration of headache attributed to COVID-19 vaccination (h)	13.6 ± 17.1 n = 210	14.2 ± 21.1 *n* = 1177	*P = 0.695*
Intensity of headache attributed to COVID-19 vaccination (VRS 0–5)	3.3 ± 0.9 *n* = 272	3.3 ± 0.9 *n* = 1462	*P = 0.419*

## Discussion

Headaches after COVID-19 vaccination show an extensive complex of symptoms. The constellation of accompanying symptoms together with the temporal and spatial headache characteristics delimit a distinctive headache phenotype for headaches after COVID-19 vaccination with the BNT162b2 mRNA COVID-19 vaccine. They occur with similar symptoms in all age groups. Less than 10% of those affected also report headaches resulting from previous vaccinations. And of these, 84.9% report that the headache after COVID-19 vaccination is not similar to the headache following previous other vaccinations. The headache after a COVID-19 vaccination occurs on average around 18 h after vaccination. It has an average duration of 14 h. In around two-thirds of those affected the headache lasts between 1 and 12 h. Over 66% experience a monophasic headache course. The headache usually occurs bilaterally, the main localization being forehead, temples, back of the head and retroorbital region. They do not radiate to other regions. The pain character is most frequently described as pressing and dull. The pain intensity is moderate to severe. Routine physical activity can aggravate the headache. The most common accompanying symptoms are fatigue, exhaustion and muscle pain. These can be accompanied by nausea as well as hypersensitivity to light and noise. In women, the duration and the intensity of headache are significantly greater than in men. This analysis does not aim to attribute a cause to each individual symptom. The aim of the study is to record the headache symptom complex in temporal relation to the vaccination. With regard to the vaccine type used for the very first time, the aim was to determine this complex of symptoms in detail.

This analysis has several limitations. By voluntarily participating in the study, it is possible that participants with particularly pronounced headaches preferentially participated. The resulting clinical headache phenotype could thus describe particularly severe forms. We have, therefore, tried to capture the headache phenotype in the most comprehensive way possible by recruiting a very large number of participants. Regarding the gender ratio, the study does not allow a definitive statement. Since the population of the vaccinees is not known and study participation was voluntary, it is possible that, given a disproportionate proportion of female vaccinees in the study, an excessive number of headache sufferers from the group of women participated in the study. Since headaches occur very frequently, regardless of vaccination, the data may be confounded by these other headaches. However, the resulting headache phenotype is neither that of a migraine nor that of a tension-type headache. It is possible that clinical symptoms of these two primary forms of headache are included in the reports and overlap with the headache phenotype from COVID-19 vaccination. Nevertheless, the described symptom complex of headache after COVID-19 vaccination stands out from the above-mentioned primary headaches.

This report does not address the frequency of headache occurrences after COVID-19 vaccination. The Phase 3 clinical trials of the various vaccination candidates provide detailed information about this by recording the frequency of possible side effects of the vaccination.^5–9^^,^^13^^–^^20^ On the other hand, no detailed information regarding the clinical phenotype is known from these studies. Whether the headache phenotype described here occurs in the same way or in a different form with other vaccines against COVID-19 is subject of further studies. The questionnaire does not contain any mandatory questions. Therefore, detailed information on some individual variables such as age, for example, is not available for the entire sample.

The International Classification of Headache Disorders^12^ lists headaches attributed to intracranial and systemic infections either bacterial, viral, fungal or parasitic. Headaches attributed to systemic viral infection are also described. A specific headache phenotype is not described in these cases. Headaches after vaccination are not yet listed in the International Classification of Headache Disorders. In particular, no detailed knowledge exists about the form of headaches occurring after a vaccination with an mRNA vaccine in general and specifically with the BNT162b2 mRNA COVID-19 vaccine. The findings described in this report for the first time provide an overview of the phenotype of this headache, which can occur in more than 50% of the participants after vaccination as the most frequent neurological symptom.

Knowledge of this phenotype is important because these headaches can occur in patients who otherwise have no other headache conditions. It helps to differentiate these headaches from other spontaneously occurring headache causes. While headaches in relation to a systemic viral infection typically have no specific headache characteristics with regard to the temporal aspects, the pain character, the location and the accompanying symptoms, the findings of this study delineate a distinctive headache symptom complex with characteristic accompanying symptoms.

Whether and in what form after the acute disappearance of headaches after vaccination non-headache symptoms occur in the postdrome^21^ is not yet known. In this first study to describe the headache after vaccination, the acute phase should initially be described. Postdromal symptoms need to be investigated in further studies.

According to the International Classification of Headache Disorders, 3rd Edition,^12^ headaches that are attributed to a systemic viral infection require diagnostic evidence of a systemic viral infection without evidence of meningitis or encephalitis. The headache must be closely related to the onset of the viral infection, aggravate as the viral infection worsens, and improve as the viral infection disappears. The pain is described as diffuse and of moderate or severe intensity. Headache after vaccination against COVID-19 with the mRNA vaccine is not due to a systemic viral infection. For the first time, the exact time between vaccination and the onset of the headache, the duration of the headache, the headache character, the location and specific accompanying symptoms are described in this study. These characteristics have not yet been included in the International Classification of Headache Disorders.

The pathomechanisms of headache after vaccination against COVID-19 are not yet understood. It must remain open at present, whether the spike protein, synthesized intracellularly using the mRNA supplied by the vaccine, is itself responsible for the headache or whether it is due to the resulting immune response triggered by that protein.^5^^,^^22–27^ The intracellular formation of the spike protein and the immune response triggered by it could be directly related to the development of the headache phenotype described, including the accompanying symptoms of tiredness, exhaustion, muscle pain, dizziness, poor concentration, chills and fever. It is speculated that microorganisms can activate anti-inflammatory substances, such as nitric oxide, prostaglandins and cytokines.^22^^,^^28^ Extensive amounts of proinflammatory cytokines can be released during a COVID-19 infection. Also, according to our results, 11.1% of subjects with headache after vaccination reported fever as an accompanying symptom. It can therefore be assumed that inflammatory mediators are involved in the development of headaches after COVID-19 vaccination as well at least in patients who developed fever.

Pre-existing primary headaches such as migraine lead to an increased duration and pain intensity of the headache after COVID-19 vaccination. It is possible that the sensitization with hyperexcitability of trigeminovascular neurons, existing in primary headaches, causes an increase in pain sensitivity and that this is relevant for the intensification of headaches after COVID-19 vaccination.

In conclusion, headaches after COVID-19 vaccination show concise clinical characteristics. The constellation of accompanying symptoms together with the temporal and spatial headache characteristics delimit a distinctive headache phenotype for headaches after COVID-19 vaccination with the BNT162b2 mRNA COVID-19 vaccine. It must remain open at present, whether the spike protein, synthesized intracellularly using the mRNA supplied by the vaccine, is itself responsible for the headache or whether it is due to the resulting immune response triggered by that protein. Whether the headache phenotype described here occurs in the same way or in a different form with other vaccines against COVID-19 is subject of further studies.^29^

## Funding

The authors acknowledge financial support by the state of Schleswig-Holstein within the funding programme Open Access Publication Fund.

## Competing interests

The authors report no competing interests.

## Abbreviations

COVID-19 =: coronavirus disease 2019
SARS-CoV-2 =: severe acute respiratory syndrome coronavirus type 2
VRS =: Verbal Rating Scale
